# Racial and Sex Disparities in Gout Prevalence Among US Adults

**DOI:** 10.1001/jamanetworkopen.2022.26804

**Published:** 2022-08-15

**Authors:** Natalie McCormick, Na Lu, Chio Yokose, Amit D. Joshi, Shanshan Sheehy, Lynn Rosenberg, Erica T. Warner, Nicola Dalbeth, Tony R. Merriman, Kenneth G. Saag, Yuqing Zhang, Hyon K. Choi

**Affiliations:** 1 Clinical Epidemiology Program, Division of Rheumatology, Allergy, and Immunology, Massachusetts General Hospital, Boston; 2 Mongan Institute, Department of Medicine, Massachusetts General Hospital, Boston; 3 Department of Medicine, Harvard Medical School, Boston, Massachusetts; 4 Arthritis Research Canada, Vancouver, British Columbia; 5 Clinical and Translational Epidemiology Unit, Massachusetts General Hospital, Boston; 6 Slone Epidemiology Center at Boston University, Boston, Massachusetts; 7 Harvard/MGH Center on Genomics, Vulnerable Populations, and Health Disparities, Boston, Massachusetts; 8 Department of Medicine, University of Auckland, Auckland, New Zealand; 9 Division of Clinical Immunology and Rheumatology, University of Alabama at Birmingham; 10 Department of Biochemistry, University of Otago, Dunedin, New Zealand

## Abstract

**Importance:**

Emerging data suggest gout and hyperuricemia may now be more frequent among Black adults in the US than White adults, especially Black women. However, national-level, sex-specific general population data on racial differences in gout prevalence and potential socioclinical risk factors are lacking.

**Objective:**

To identify sex-specific factors driving disparities between Black and White adults in contemporary gout prevalence in the US general population.

**Design, Setting, and Participants:**

This cross-sectional analysis used nationally representative, decadal survey data from successive cycles of the National Health and Nutrition Examination Survey from 2007 to 2016. Data were analyzed from November 1, 2019, through May 31, 2021. Participants included US adults self-reporting Black or White race.

**Exposures:**

Self-reported race, excess body mass index, chronic kidney disease (CKD; defined as estimated glomerular filtration rate <60 mL/min/1.73 m^2^, according to latest equations without race coefficient), poverty, poor-quality diet, low educational level, alcohol consumption, and diuretic use.

**Main Outcomes and Measures:**

Race- and sex-specific prevalence of physician- or clinician-diagnosed gout and hyperuricemia and their differences before and after adjusting for potential socioclinical risk factors.

**Results:**

A total of 18 693 participants were included in the analysis, consisting of 3304 Black women (mean [SD] age, 44.8 [0.4] years), 6195 White women (mean [SD] age, 49.8 [0.3] years), 3085 Black men (mean [SD] age, 43.6 [0.5] years]), and 6109 White men (mean [SD] age, 48.2 [0.3] years). Age-standardized prevalence of gout was 3.5% (95% CI, 2.7%-4.3%) in Black women and 2.0% (95% CI, 1.5%-2.5%) in White women (age-adjusted odds ratio [OR], 1.81 [95% CI, 1.29-2.53]); prevalence was 7.0% (95% CI, 6.2%-7.9%) in Black men and 5.4% (95% CI, 4.7%-6.2%) in White men (age-adjusted OR, 1.26 [95% CI, 1.02-1.55]). These associations attenuated after adjusting for poverty, diet, body mass index, and CKD among women and for diet and CKD among men but became null after adjusting for all risk factors (ORs, 1.05 [95% CI, 0.67-1.65] among women and 1.05 [95% CI, 0.80-1.35] among men). Hyperuricemia end point findings were similar.

**Conclusions and Relevance:**

In this nationally representative race- and sex-specific cross-sectional study of US adults, gout was more prevalent in adults self-reporting Black race during a recent 10-year period compared with their White counterparts. These racial differences may be explained by sex-specific differences in diet and social determinants of health and clinical factors. Culturally informed efforts focusing on these factors could reduce current gout-related disparities.

## Key Points

**Question:**

Are there racial differences in gout prevalence among adults in the US general population, and what might explain these disparities?

**Findings:**

In this cross-sectional study of 18 693 participants, gout was 1.8 times more prevalent among Black women than White women and 1.3 times more prevalent among Black men than White men. These associations attenuated most after adjusting for poverty, diet, body mass index, and chronic kidney disease (CKD) among women and for diet and CKD among men; they were nullified in both sexes after adjusting for all risk factors, with similar findings for hyperuricemia.

**Meaning:**

These findings suggest that racial disparities in gout may be explained by diet, social determinants of health, and CKD, which could help identify interventions to reduce these disparities.

## Teaser

This cross-sectional study investigates racial differences in gout prevalence among women and men in the US general population and identifies sex-specific factors that may explain these differences.

## Introduction

Gout has historically been considered a disease of White men who overindulge in gamey meats and other rich foods.^1^ However, emerging data suggest gout and hyperuricemia (its causal precursor) impart an even larger burden on other demographic groups, including Black men, Black women, and White women.^2^ Indeed, the global frequency and disability burden of gout among women have been rising disproportionately relative to gout among men,^3^ with gout among women characterized by a higher frequency of obesity^4,5^ and related cardiometabolic sequalae.^4,5,6,7^ At the same time, previous studies have suggested that compared with White US residents, Black US residents are at increased risk for gout^2,8,9^ and lower health-related quality of life.^10^ However, national-level general population data on sex-specific racial differences in the burden of gout are lacking.

Importantly, potential racial differences may be largely attributable to differences in nongenetic social determinants of health^11^ rather than ancestry-specific differences in genes regulating urate handling.^12^ For example, excess adiposity, a major risk factor for gout^13,14^ and hyperuricemia,^15,16,17^ is more prevalent among Black women in the US general population than White women.^18,19,20,21^ Moreover, after accounting for body mass index (BMI), alcohol consumption, and other factors, Black men had a lower risk of incident gout and incident hyperuricemia in the all-male Multiple Risk Factor Intervention Trial.^11^ However, general population data are lacking for both women and men; sex-specific analysis is critical given that women and men tend to differ in their gout risk factor profiles (eg, higher frequencies of diuretic use^5,7,22^ and obesity^4,5^ and lower frequency of alcohol consumption^4,5^ have been observed in women vs men).

Our objective was to investigate current racial differences in gout prevalence among women and men in the US general population and identify sex-specific factors that may explain these differences. We hypothesized these differences would be present at the national level and would be associated, in a sex-specific manner, with racial differences in social and lifestyle factors, diuretic use, and chronic kidney disease (CKD).

## Methods

### Data Source

The National Health and Nutrition Examination Survey (NHANES) is a cross-sectional, nationwide survey in the US that assesses the health and nutritional status of adults and children using interviews, physical examinations, and laboratory data.^23^ NHANES uses a complex, multistage probability design to provide a nationally representative sample of the noninstitutionalized US civilian population. The present study included participants 18 years or older with data on gout status and serum urate levels using data collected from the 2007-2008 through 2015-2016 annual NHANES cycles; gout diagnoses were not ascertained in the earlier surveys (1999-2000 through 2005-2006). However, data on gout diagnoses were ascertained in the NHANES III (1988-1994) and were used to compare the current sex- and race-specific estimates with those from more than 2 decades earlier. All procedures in each NHANES were approved by the National Center for Health Statistics Ethics Review Board, and written informed consent was obtained from participants at the time of enrollment. This study followed the Strengthening the Reporting of Observational Studies in Epidemiology (STROBE) reporting guideline for cross-sectional studies.

During the in-person interviews, participants reported on their age, sex, race and ethnicity, educational attainment, household size and income, recent dietary intake, medical history (including gout), and use of prescription medications within the past 30 days (including diuretics). Race and ethnicity were categorized as Hispanic (Mexican and non-Mexican Hispanic), non-Hispanic Asian (from 2011 onward), non-Hispanic Black or African American (hereafter referred to as Black), non-Hispanic White (hereafter referred to as White), and other race and ethnicity; the present analysis was limited to those self-identifying as Black or White race. Household income and size were used to determine the poverty to income ratio, a derived variable used in prior NHANES studies^24,25,26^ and calculated as household income, adjusted for household size, divided by the year-specific poverty guideline^27^ issued by the US Department of Health and Human Services. These guidelines are used to determine eligibility for many federal benefit programs, including the Supplemental Nutrition Assistance Program.^24,25,26,28^ Alcoholic beverage consumption (number of drinks per week) was computed from responses to questions on the frequency of alcoholic beverage consumption (eg, number of days per week, month, or year) and total number of drinks per day of consumption. Two 24-hour dietary recalls were undertaken to ascertain the types and amounts of all foods and beverages consumed by participants. After mapping to the US Department of Agriculture Food Patterns Equivalents Database,^29^ these data were used to compute a diet quality score based on the dietary components emphasized or minimized in the Dietary Approaches to Stop Hypertension (DASH) diet.^30,31,32^

At the mobile examination units, participants had their height and weight measured by trained staff using standardized instruments, with BMI calculated by dividing the weight in kilograms by the height in meters squared. Blood was drawn for measurement of serum levels of urate, creatinine, and other biomarkers. Serum creatinine levels were used to determine glomerular filtration rate according to the latest equations, which do not include a coefficient for Black race.^33^

### Assessment of Gout and Hyperuricemia

During the NHANES home interviews, all participants were asked, “Has a doctor or other health professional ever told you that you had gout?” Serum urate levels were measured in blood samples obtained at the time of enrollment using a colorimetric method where urate is oxidized to allantoin and hydrogen peroxide by uricase using a multichannel analyzer (Hitachi Model 737; Boehringer Mannheim Diagnostics). The quality-control procedures have been described elsewhere.^34^ Values are reported in milligrams per deciliter and can be converted to micromoles per liter by multiplying by 59.48. Hyperuricemia was defined according to the top 10th percentile of serum urate levels for women (6.8 mg/dL) and men (7.8 mg/dL), considering the biological urate saturation point as well as statistical efficiency.

### Potential Risk Factors

Potential risk factors, which were selected on the basis of prior knowledge,^13,14,31,32,35,36,37,38,39^ included low educational level (high school or less [dichotomous]), poverty (household income <130% of the federal poverty guideline [dichotomous]),^25^ BMI (continuous),^13,14,40,41^ number of alcoholic beverages consumed per week (continuous), poor diet quality (lower level of adherence to the DASH pattern [continuous]),^31,32^ diuretic use in the past 30 days (dichotomous),^38,39,40^ and CKD (estimated glomerular filtration rate <60 mL/min/1.73 m^2^ [dichotomous]).^36^ To enhance comparability, factors were scored such that their presence (or higher values) corresponded to a greater risk of gout. For example, the continuous DASH score was inverted such that higher scores indicated lower levels of adherence.

### Statistical Analysis

Data were analyzed from November 1, 2019, through May 31, 2021. Incorporating sample weights and accounting for the clusters and strata of the complex study design of the NHANES, we calculated the prevalence of gout and hyperuricemia within the US adult Black and White populations according to sex. We next determined the association between each risk factor and the odds of gout among men and women, adjusting for age and race, along with racial differences in the levels and frequencies of each risk factor (specifically, the age-adjusted difference between Black and White women and Black and White men). We then performed stepwise logistic regression by adding potential risk factors one by one to show how the association between self-reported Black race and gout changed with the addition of each variable, among those with complete data available, for women and men separately. We started with educational level, followed by poverty (2 social factors), then consecutively added alcohol consumption, diet score, and BMI (3 lifestyle factors potentially associated with educational level and poverty), and finished with diuretic medication use and CKD status (2 clinical factors potentially associated with the other purported risk factors). Characteristics of these women and men were similar to those of the sample at large (eTable 1 in the Supplement), as was age-adjusted prevalence of gout and hyperuricemia (eTable 2 in the Supplement). We calculated 95% CIs for all effect estimates. We used SAS, version 9.4 (SAS Institute, Inc) for all statistical analyses. Two-sided *P* < .05 indicated statistical significance.

## Results

Overall, we included 18 693 participants comprising 3085 Black men (mean [SD] age, 43.6 [0.5] years), 3304 Black women (mean [SD] age, 44.8 [0.4] years), 6109 White men (mean [SD] age, 48.2 [0.3] years), and 6195 White women (mean [SD] age, 49.8 [0.3] years). Table 1 shows the characteristics stratified by sex and race. For both sexes, Black participants tended to be younger than White participants and had a higher frequency of poverty and lower educational attainment. With the exception of alcohol consumption, which was greater among White adults, all risk factors for gout and hyperuricemia were more prevalent in Black adults than White adults.

**Table 1.** Participant Characteristics by Sex and Race

Characteristic	Women		Men	
	Black (n = 3304)	White (n = 6195)	Black (n = 3085)	White (n = 6109)
Age, mean (SD), y	44.8 (0.4)	49.8 (0.3)	43.6 (0.5)	48.2 (0.3)
Educational level high school or less^a^				
No. of participants	1521	2447	1699	2612
Weighted % (95% CI)	43.5 (40.8-46.3)	32.7 (30.0-35.4)	52.9 (50.2-55.6)	35.4 (32.2-38.6)
Family income-to-poverty ratio <1.3^a^				
No. of participants	1694	1201	904	1542
Weighted % (95% CI)	39.7 (36.2-43.1)	18.0 (16.0-20.0)	32.4 (29.3-35.4)	14.8 (13.0-16.6)
BMI, mean (SD)	32.0 (0.2)	28.7 (0.1)	28.9 (0.1)	28.7 (0.1)
No. of alcoholic drinks consumed per week, mean (SD)	2.0 (0.1)	2.4 (0.1)	5.1 (0.2)	5.7 (0.2)
DASH score, mean (SD)^b^	28.9 (0.1)	25.3 (0.1)	30.4 (0.1)	27.4 (0.1)
Diuretic use^a^				
No. of participants	419	639	293	540
Weighted % (95% CI)	10.8 (9.7-11.9)	8.4 (7.6-9.2)	6.8 (5.8-7.9)	6.1 (5.5-6.8)
CKD (eGFR <60 mL/min/1.73 m^2^)^a^				
No. of participants	347	596	299	479
Weighted % (95% CI)	9.6 (8.4-10.7)	7.3 (6.6-8.0)	7.6 (6.7-8.4)	4.9 (4.3-5.6)

Abbreviations: BMI, body mass index (calculated as weight in kilograms divided by height in meters squared); CKD, chronic kidney disease; DASH, Dietary Approaches to Stop Hypertension; eGFR, estimated glomerular filtration rate.

^a^ Numbers of individuals included in the sample are unweighted; percentages represent the weighted frequency of the risk factor in the corresponding stratum of the US population.

^b^ All factors were scored such that their presence (higher values) corresponded to a greater risk of gout or hyperuricemia. Therefore, the continuous DASH score was inverted such that higher scores indicate lower levels of adherence to a DASH-style diet.

The age-adjusted prevalence of gout during the 2007-2016 study period was 7.0% (95% CI, 6.2%-7.9%) in Black men, 3.5% (95% CI, 2.7%-4.3%) in Black women, 5.4% (95% CI, 4.7%-6.2%) in White men, and 2.0% (95% CI, 1.5%-2.5%) in White women (Table 2). Black race was associated with greater odds of gout during this period among women (odds ratio [OR], 1.81 [95% CI, 1.29-2.53]), among men (OR, 1.26 [95% CI, 1.02-1.55]) (Table 2), and overall (OR, 1.46 [95% CI, 1.22-1.74]). In contrast, there was no racial difference in the prevalence of gout approximately 2 decades earlier in the 1988-1994 NHANES III (eFigure in the Supplement), with age-adjusted ORs of 0.98 (95% CI, 0.65-1.47) among women, 0.91 (95% CI, 0.68-1.21) among men, and 0.93 (95% CI, 0.73-1.17) overall.

**Table 2.** Sex-Specific Prevalence of Gout and Hyperuricemia and Estimated Annualized Number of Affected Black and White Adults in the US Population^a^

Stratification by sex and race	Age-adjusted outcome					
	Gout			Hyperuricemia		
	Prevalence, % (95% CI)	No. of US adults, 1 million	OR (95% CI)	Prevalence, % (95% CI)	No. of US adults, 1 million	OR (95% CI)
Women						
Black	3.5 (2.7-4.3)	0.5	1.81 (1.29-2.53)	10.5 (9.2-11.8)	1.3	2.00 (1.62-2.47)
White	2.0 (1.5-2.5)	1.6	[Reference]	5.6 (4.8-6.3)	4.0	[Reference]
Men						
Black	7.0 (6.2-7.9)	0.8	1.26 (1.02-1.55)	11.0 (9.9-12.1)	1.1	1.39 (1.15-1.68)
White	5.4 (4.7-6.2)	3.9	[Reference]	7.8 (6.9-8.8)	4.9	[Reference]

Abbreviation: OR, odds ratio.

^a^ Data are from the 2007-2008 through 2015-2016 National Health and Nutrition Examination Surveys.

### Racial Differences Among Women With Gout

All risk factors except educational level were associated with gout among women in age- and race-adjusted analysis (eTable 3 in the Supplement). Notably, poverty was associated with 2.4 times greater odds of gout (OR, 2.36 [95% CI, 1.79-3.10]), whereas current use of diuretic medications was associated with 2.2 times greater odds of gout (OR, 2.17 [95% CI, 1.59-2.95]). Furthermore, all deleterious factors were more frequent (or levels were elevated) in Black women compared with White women, except for alcohol consumption, which was lower among Black women (eTable 3 in the Supplement). For example, age-adjusted mean BMI was higher in Black women (difference, 3.2 [95% CI, 2.9-3.6]) than White women, and a greater proportion of Black women than White women had incomes below the poverty guideline (difference, 8.4% [95% CI, 6.0%-10.7%]). In our stepwise regression analysis (Table 3), the greatest attenuations in the association between self-reported race and gout occurred after adjustment for poverty, diet, BMI, and CKD; adjustment for CKD, the final variable, rendered a null OR of 1.05 (95% CI, 0.67-1.65).

**Table 3.** Stepwise Regression for Potential Risk Factors for the Association Between Black Race and Odds of Gout and Hyperuricemia Among Women^a^

Factor	Black vs White, OR (95% CI)	
	Gout	Hyperuricemia
Age-adjusted only	1.60 (1.05-2.44)	1.98 (1.58-2.49)
With educational level added^b^	1.58 (1.04-2.41)	1.90 (1.51-2.38)
With poverty added^c^	1.33 (0.88-2.01)	1.71 (1.36-2.15)
With alcohol consumption added^d^	1.34 (0.88-2.02)	1.72 (1.37-2.17)
With DASH diet score added^e^	1.28 (0.83-1.97)	1.52 (1.19-1.93)
With BMI added^f^	1.10 (0.72-1.69)	1.22 (0.96-1.57)
With diuretic use added^g^	1.08 (0.70-1.68)	1.19 (0.93-1.53)
With CKD added^h^	1.05 (0.67-1.65)	1.01 (0.76-1.35)

Abbreviations: BMI, body mass index; CKD, chronic kidney disease; DASH, Dietary Approaches to Stop Hypertension; OR, odds ratio.

^a^ Data are from the 2007-2008 through 2015-2016 National Health and Nutrition Examination Surveys. Estimates for each factor were generated from sample with complete data on all variables.

^b^ Dichotomized as high school graduate or less vs some college or more.

^c^ Dichotomized as family income-to-poverty ratio less than 1.3 (household income <130% of the federal poverty guideline) vs 1.3 or greater.

^d^ Continuous variable measured as number of alcoholic drinks consumed per week.

^e^ Continuous variable (range, 9-45); higher scores reflect lower DASH-style diet adherence.

^f^ Continuous variable; higher values are associated with greater adiposity.

^g^ Dichotomized as yes or no.

^h^ Dichotomized as estimated glomerular filtration rate less than 60 vs at least 60 mL/min/1.73 m^2^.

### Racial Differences Among Men With Gout

Most purported risk factors were associated with gout among men, including alcohol consumption, although unlike gout among women, the association with poverty was not significant (OR, 1.14 [95% CI, 0.93-1.40]) (eTable 4 in the Supplement). As with women, alcohol consumption was lower among Black men than White men, but the racial differences in BMI and poverty status between Black and White men were smaller than those between Black and White women. As such, adjustment for poverty and BMI did not eliminate the association between race and gout in the stepwise regression analysis (Table 4), whereas adjustment for CKD rendered a null OR of 1.05 (95% CI, 0.80-1.35).

**Table 4.** Stepwise Regression for Potential Risk Factors for the Association Between Black Race and Odds of Gout and Hyperuricemia Among Men^a^

Factor	Black vs White, OR (95% CI)	
	Gout	Hyperuricemia
Age-adjusted only	1.28 (1.01-1.62)	1.42 (1.14-1.76)
With educational level added^b^	1.26 (0.98-1.61)	1.42 (1.14-1.76)
With poverty added^c^	1.23 (0.97-1.55)	1.41 (1.13-1.75)
With alcohol consumption added^d^	1.24 (0.98-1.57)	1.42 (1.14-1.77)
With DASH diet score added^e^	1.16 (0.91-1.48)	1.37 (1.09-1.72)
With BMI added^f^	1.15 (0.90-1.47)	1.31 (1.03-1.67)
With diuretic use added^g^	1.14 (0.89-1.46)	1.24 (0.96-1.61)
With CKD added^h^	1.05 (0.80-1.35)	1.08 (0.81-1.42)

Abbreviations: BMI, body mass index; CKD, chronic kidney disease; DASH, Dietary Approaches to Stop Hypertension; OR, odds ratio.

^a^ Data are from the 2007-2008 through 2015-2016 National Health and Nutrition Examination Surveys. Estimates for each factor generated from sample with complete data on all variables.

^b^ Dichotomized as high school graduate or less vs some college or more.

^c^ Dichotomized as family income-to-poverty ratio less than 1.3 (household income <130% of the federal poverty guideline) vs 1.3 or greater.

^d^ Continuous variable measured as number of alcoholic drinks consumed per week.

^e^ Continuous variable (range, 9-45); higher scores reflect lower DASH-style diet adherence.

^f^ Continuous variable; higher values are associated with greater adiposity.

^g^ Dichotomized as yes or no.

^h^ Dichotomized as estimated glomerular filtration rate less than 60 vs at least 60 mL/min.

### Racial Differences in Hyperuricemia

The stepwise regression analysis for racial differences in hyperuricemia prevalence followed closely consistent patterns of gout among men and women (Tables 3 and 4), with the greatest attenuations in the association for hyperuricemia among women occurring after adjustment for poverty (OR, 1.71 [95% CI, 1.36-2.15]), BMI (OR, 1.22 [95% CI, 0.96-1.57]), diet (OR, 1.52 [95% CI, 1.19-1.93]), and CKD (OR, 1.01 [95% CI, 0.76-1.35]). Among men, the greatest attenuations were observed after adjustment for diuretic use (OR, 1.24 [95% CI, 0.96-1.61]) and CKD (OR, 1.08 [95% CI, 0.81-1.42]).

## Discussion

In this nationally representative, race- and sex-specific study of US adults, gout was more prevalent in adults who self-reported Black race during a recent 10-year period compared with their White counterparts. This disparity is in contrast with data from 2 decades earlier (the NHANES III), when no differences in gout prevalence were observed between Black and White adults, and parallels a disproportionate worsening of CKD^42^ and overweight and obesity^21^ among Black adults over time. Indeed, our findings suggest that the current racial disparities (2007-2016) may be explained by differences in the frequencies or levels of key social or lifestyle and clinical factors. Importantly, there were distinct sex-specific patterns, with higher BMI levels and poverty rates among Black vs White women than among Black vs White men. At the same time, alcohol consumption, an established gout risk factor, was greater in White adults than in Black adults. These findings show that gout is no longer exclusive to affluent White men (ie, the “disease of kings”)^43^—if it ever was—while highlighting social determinants of health that could serve as targets for reducing these disparities in the general population.

Excess adiposity is a foremost risk factor for gout^13,41^ and hyperuricemia,^15^ and in the present study, where Black women had higher BMI levels than White women, adjustment for BMI attenuated much of the racial difference in gout and hyperuricemia among women. Poverty and poor-quality diet, factors that may themselves affect BMI, were already present in the model. Higher BMI levels among Black women than White women were also reported in previous NHANES^18,20^ and other US studies.^2,44,45,46^ In contrast to the findings among women, our study and previous studies^2,18,44,45,46^ found that BMI levels were similar (or even lower) among Black men compared with White men and that adjustment for BMI did not eliminate the association between race and gout in men. Adiposity increases serum urate levels and gout risk by reducing urate excretion and increasing urate production.^47,48,49,50,51,52^ Ancillary analysis of a randomized dietary intervention trial showed healthy weight loss diets could reduce serum urate levels, especially among those with baseline hyperuricemia,^53^ whereas bariatric surgery has been associated with reductions in serum urate levels and incidence of gout and hyperuricemia.^54,55^ Furthermore, a recent sex-specific cohort study^13^ found that excess adiposity was associated with risk of incident gout among women and men, corroborating the larger effect size of BMI that we observed among women. Together with the findings of the present study, individual- and societal-level strategies to help Black women achieve and maintain a healthy body weight may help reduce disparities in the incidence of gout among Black and White women.

Another key risk factor, CKD, also attenuated the racial differences for gout and hyperuricemia, particularly in men, even with prior adjustment for documented CKD risk factors, including adiposity, poverty,^56^ and DASH adherence.^57^ Although the higher prevalence of CKD in Black adults (compared with White adults) has been attributed in part to the *APOL1* risk alleles present in African American populations,^58,59^ evidence suggests that racial differences in sociodemographic, lifestyle, and clinical factors play a greater role,^60^ with poorer access to health care among Black individuals also contributing.^61^

Although gout has historically been viewed as a disease of wealthy White men who could afford to overindulge in alcohol and purine-rich foods, the association between socioeconomic status and gout and hyperuricemia has garnered little empirical study and remains unclear.^62^ Our contemporary findings show that poverty (ie, low household income level) is associated with more than 2 times greater odds of gout among women (OR, 2.36 [95% CI, 1.79-3.10]) and may explain a portion of the racial differences among women with gout, whereas there was no association between poverty and gout among men (OR, 1.14 [95% CI, 0.93-1.40]). Although this may reflect the excess burden of gout-related cardiometabolic and kidney sequalae among lower-income individuals,^24,63^ given the associations among socioeconomic status, food insecurity, diet quality, and obesity,^25,64^ especially among women,^65^ poverty could have downstream associations with diet and BMI.

The serum urate level–lowering effects of DASH-style diets (vs typical US diets) have been demonstrated in randomized clinical trials,^66,67^ whereas prospective cohort studies have reported inverse associations between DASH adherence (adjusted for total energy intake) and the clinical end point of incident gout.^31,32^ However, greater DASH adherence is also associated with higher food costs^68,69^; US adults living in poverty have had lower DASH adherence scores than those with higher incomes,^57,68^ as have Black adults compared with White adults,^57,68^ with evidence of higher relative costs to achieve DASH adherence among Black adults than White adults.^68^ As such, interventions aimed at promoting healthy eating patterns such as DASH or others described in the Dietary Guidelines for Americans,^31^ and reducing barriers^70^ to adherence, including for those receiving Supplemental Nutrition Assistance Program benefits (who tend to have poorer-quality diets even compared with eligible individuals not receiving benefits),^71^ could reduce racial disparities in the prevalence of gout and hyperuricemia, particularly among women.

Diuretic medication use also contributed to racial differences in both gout and hyperuricemia, with significantly more Black women and men currently using diuretics than their White counterparts. This is likely related to higher frequency of hypertension among Black individuals, particularly among Black women,^72^ although choice of antihypertensive agent may vary as well. Antihypertensive agents that lower urate levels (eg, calcium channel blockers or losartan) could be preferred to minimize gout risk,^38,73^ whereas potassium-sparing diuretics^39^ could also be considered for individuals at risk of gout when clinically appropriate. Finally, the inverse racial difference in levels of alcohol consumption is consistent with studies reporting higher levels of alcohol consumption among White women and men than their Black counterparts.^74^

### Strengths and Limitations

The present study had several strengths. Given that NHANES data are collected from community-based samples and weighted to be representative of the US population, our findings are generalizable to Black and White individuals in the US at large. Although the self-reported physician- and clinician-sourced diagnoses of gout were not clinically confirmed, this definition performed well in a validation study,^75^ and residual misclassification would likely bias our estimates toward the null. Furthermore, our findings for gout were consistent with those for hyperuricemia, an objective laboratory end point.

This study also has limitations. Our study was cross-sectional, although the plausible temporal associations among race, risk factors, and outcomes were apparent, and any residual plausible reverse causations would likely have made our effect estimates conservative (eg, known gout leading to diet change). The associations between risk factors and outcomes were also consistent with those reported for incident outcomes in prospective cohort studies.^13,14,31,32,35,36,37,38,39^ Nevertheless, as with any observational study, our analyses may be subject to residual or unmeasured confounding. Finally, we acknowledge that the racial differences reported herein may be influenced by racism^76^ and other resultant factors not explicitly included in our models, such as stress, lower levels of physical activity, and poorer access to health care. Importantly, however, the investigated factors, which together nullified the racial differences in gout among men and women, are likely correlated with these and other key contributors. For example, the built environment in some poorer neighborhoods may pose barriers to physical activity, which has implications for BMI, whereas clinician bias and intergenerational mistrust can result in Black individuals receiving lower-quality health care, which has implications for the development of CKD.^61^

## Conclusions

The findings of this nationally representative, cross-sectional study of gout prevalence in US adults suggest that gout may be more common in Black men and women compared with their White counterparts. Moreover, these differences may be explained by racial differences in key socioclinical factors, including excess BMI, poverty, and poor diet as well as CKD in women and by CKD, poor diet, and diuretic use in men. Culturally informed interventions designed to address adiposity and kidney disease and improve diet quality while recognizing the role of poverty in gout among women could help reduce these disparities.

## Notes

Supplement. **eTable 1.** Participant Characteristics by Sex and Race (Complete Case Analysis) **eTable 2.** Sex-Specific Association Between Black Race and Gout and Hyperuricemia, 2007-2008 to 2015-2016 NHANES (Complete Case Analysis) **eTable 3.** Association of Risk Factors With Gout and Hyperuricemia Among Women **eTable 4.** Association of Risk Factors With Gout and Hyperuricemia Among Men **eFigure.** Age-Adjusted Prevalence of Gout Among Black and White US Adults Between NHANES III (1988-1994) and NHANES 2006-2017
