# Supplementary material for: Racial and Sex Disparities in Gout Prevalence Among US Adults
Source: JAMA Netw Open. 2022 Aug 15;5(8):e2226804. doi: 10.1001/jamanetworkopen.2022.26804 (PMC9379746; doi:10.1001/jamanetworkopen.2022.26804)
Supplement: Supplement. — eTable 1. Participant Characteristics by Sex and Race (Complete Case Analysis) eTable 2. Sex-Specific Association Between Black Race and Gout and Hyperuricemia, 2007-2008 to 2015-2016 NHANES (Complete Case Analysis) eTable 3. Association of Risk Factors With Gout and Hyperuricemia Among Women eTable 4. Association of Risk Factors With Gout and Hyperuricemia Among Men eFigure. Age-Adjusted Prevalence of Gout Among Black and White US Adults Between NHANES III (1988-1994) and NHANES 2006-2017 [file jamanetwopen-e2226804-s001.pdf]

Supplementary Online Content

McCormick N, Lu N, Yokose C, et al. Racial and sex disparities in gout prevalence among US adults. *JAMA Netw Open*. 2022;5(8):e2226804.
doi:10.1001/jamanetworkopen.2022.26804

eTable 1. Participant Characteristics by Sex and Race (Complete Case Analysis)
eTable 2. Sex-Specific Association Between Black Race and Gout and Hyperuricemia, 2007-2008 to 2015-2016 NHANES (Complete Case Analysis)
eTable 3. Association of Risk Factors With Gout and Hyperuricemia Among Women
eTable 4. Association of Risk Factors With Gout and Hyperuricemia Among Men
eFigure. Age-Adjusted Prevalence of Gout Among Black and White US Adults Between NHANES III (1988-1994) and NHANES 2006-2017

This supplementary material has been provided by the authors to give readers additional information about their work.

© 2022 McCormick N et al. *JAMA Network Open*.

eTable 1. Participant Characteristics by Sex and Race (complete case analysis)

	WOMEN		MEN	
	**Black (n=2,158)**	**White (n=4,764)**	**Black (n=2,037)**	**White (n=4,810)**
Age, mean (SD)	44.8 (0.5)	49.9 (0.4)	44.1 (0.5)	48.7 (0.3)
Education: high school or less, N/% (95% CI)[a]	948/41.5% (38.6% to 44.3%)	1,834/31.7% (28.9% to 34.5%)	1,095/51.6% (48.8% to 54.4%)	1,989/34.0% (30.6% to 37.4%)
Poverty: family income-to-poverty-ratio < 1.3, N/% (95% CI)[a]	796/36.7% (33.4% to 40.0%)	1,312/16.6% (14.6% to 18.7%)	626/30.9% (28.1% to 33.8%)	1,234/14.1% (12.2% to 16.0%)
BMI, mean (SD)	32.3 (0.2)	28.8 (0.1)	29.2 (0.1)	28.9 (0.1)
No. drinks per week, mean (SD)	2.0 (0.2)	2.5 (0.1)	5.1 (0.2)	5.6 (0.2)
DASH score, mean (SD)[b]	28.9 (0.1)	25.3 (0.1)	30.3 (0.2)	27.4 (0.1)
Diuretic use, N/% (95% CI)[a]	274/10.9% (9.6% to 12.2%)	470/8.1% (7.1% to 9.0%)	200/6.9% (5.8% to 8.0%)	419/6.2% (5.5% to 7.0%)
CKD: eGFR <60 mL/min, N/% (95% CI)[a]	267/9.6% (8.2% to 11.0%)	484/7.1% (6.4% to 7.9%)	221/7.2% (6.3% to 8.2%)	383/4.7% (4.0% to 5.4%)

BMI=body mass index; CKD=chronic kidney disease; DASH=Dietary Approaches to Stop Hypertension; eGFR=estimated glomerular filtration rate; SD=standard deviation; 95% CI=95% confidence interval for the weighted frequencies

[a]The N refers to the unweighted number of individuals included in the sample, whilst the % is the weighted frequency of the risk factor in the corresponding stratum of the US population.

[b]All factors were scored such that their presence (or higher values) corresponded to a greater risk of gout or hyperuricemia. Therefore, the continuous DASH score was inverted such that higher scores indicate lower levels of adherence to a DASH-style diet.

© 2022 McCormick N et al. *JAMA Network Open*.

eTable 2. Sex-Specific Association Between Black Race and Gout and Hyperuricemia, 2007-2008 to 2015-2016 NHANES (Complete Case Analysis)

	GOUT		HYPERURICEMIA	
	**Age Adjusted Prevalence, % (95% CI)**	**Age Adjusted Odds Ratio (95% CI)**	**Age Adjusted Prevalence, % (95% CI)**	**Age Adjusted Odds Ratio (95% CI)**
**Women**				
Black	3.4 (2.4 to 4.3)	1.60 (1.05 to 2.44)	10.2 (8.8 to 11.6)	1.98 (1.58 to 2.49)
White	2.1 (1.5 to 2.7)	1 [Reference]	5.6 (4.8 to 6.3)	1 [Reference]
**Men**				
Black	7.2 (6.1 to 8.3)	1.28 (1.01 to 1.62)	11.0 (9.7 to 12.3)	1.42 (1.14 to 1.76)
White	5.6 (4.8 to 6.4)	1 [Reference]	7.8 (6.7 to 8.6)	1 [Reference]

95% CI=95% confidence interval

© 2022 McCormick N et al. *JAMA Network Open*.

eTable 3. Association of Risk Factors With Gout and Hyperuricemia Among Women

Risk Factor	Exposure (Race)-Risk Factor Associations			Risk Factor-Outcome Associations*		Excess Odds Explained (%)	
	Age-Adjusted Least-Squares Mean (95% CI)[a]		Age-Adjusted Difference Between Black and White Women (95% CI)	Adjusted Odds Ratio (95% CI)[b,c]			
	Black Women	White Women		Gout	Hyperuricemia	Gout	Hyperuricemia
Education: high school or less, %	45.1 (43.0 to 47.1)	38.0 (36.6 to 39.3)	7.1 (4.6 to 9.6)	1.24 (0.94 to 1.63)	1.38 (1.16 to 1.64)	5%	4%
Poverty: family income-to-poverty-ratio < 1.3, %	36.2 (34.3 to 38.1)	27.8 (26.5 to 29.1)	8.4 (6.0 to 10.7)	2.36 (1.79 to 3.10)	1.71 (1.44 to 2.05)	26%	8%
No. drinks per week	1.9 (1.7 to 2.1)	2.4 (2.2 to 2.5)	-0.5 (-0.8 to -0.2)	1.11 (1.01 to 1.22)	1.07 (1.00 to 1.15)	-3%	-1%
DASH score (inverted)	28.4 (28.2 to 28.6)	25.6 (25.4 to 25.7)	2.9 (2.6 to 3.1)	1.20 (1.04 to 1.40)	1.26 (1.15 to 1.39)	25%	19%
BMI, kg/m^2	32.2 (31.9 to 32.5)	29.0 (28.7 to 29.2)	3.2 (2.9 to 3.6)	1.68 (1.49 to 1.89)	1.97 (1.81 to 2.13)	53%	37%
Diuretic use, %	13.9 (12.6 to 15.1)	9.3 (8.5 to 10.2)	4.5 (3.0 to 6.1)	2.17 (1.59 to 2.95)	4.11 (3.38 to 5.00)	14%	13%
CKD: eGFR <60 mL/min, %	14.2 (13.0 to 15.4)	9.3 (8.5 to 10.2)	4.8 (3.4 to 6.3)	1.71 (1.23 to 2.38)	6.40 (5.15 to 7.94)	14%	23%

[a]For dichotomous variables (Education, Poverty, Diuretic Use, and CKD), the data have been scored 0 for “no” and 1 for “yes”; therefore, the mean is equivalent to the proportion.
[b]Adjusted for age and race; [c]Difference in a dichotomous factor or 1 SD difference in a continuous factor.
BMI=body mass index; CKD=chronic kidney disease; DASH=Dietary Approaches to Stop Hypertension; eGFR=estimated glomerular filtration rate; SD=standard deviation
*Estimates for each risk factor generated from sample of women with complete data on Gout status, risk factor of interest, and all other factors. Risk Factor-Outcome Associations adjusted for age and self-reported race.

© 2022 McCormick N et al. *JAMA Network Open*.

eTable 4. Association of Risk Factors With Gout and Hyperuricemia Among Men

Risk Factor	Exposure (Race)-Risk Factor Associations			Risk Factor-Outcome Associations*		Excess Odds Explained (%)	
	Age-Adjusted Least-Squares Mean (95% CI)[a]		Age-Adjusted Difference Between Black and White men (95% CI)	Adjusted Odds Ratio (95% CI) [b,c]			
	Black men	White men		Gout	Hyperuricemia	Gout	Hyperuricemia
Education: high school or less, %	54.1 (52.0 to 56.3)	41.2 (39.8 to 42.6)	12.9 (10.3 to 15.5)	1.12 (0.93 to 1.34)	0.97 (0.83 to 1.14)	7%	-1%
Poverty: family income-to-poverty-ratio < 1.3, %	30.1 (28.2 to 32.0)	25.9 (24.7 to 27.1)	4.3 (2.0 to 6.5)	1.14 (0.93 to 1.40)	1.15 (0.96 to 1.37)	3%	2%
No. drinks per week	4.9 (4.5 to 5.3)	5.4 (5.1 to 5.6)	-0.5 (-1.0 to 0.0)	1.17 (1.08 to 1.26)	1.14 (1.07 to 1.22)	-2%	-1%
DASH score (inverted)	29.8 (29.6 to 30.0)	27.6 (27.5 to 27.7)	2.3 (2.1 to 2.5)	1.14 (1.03 to 1.25)	1.12 (1.03 to 1.22)	23%	14%
BMI, kg/m^2	29.2 (28.9 to 29.5)	28.8 (28.6 to 29.0)	0.4 (0.1 to 0.7)	1.53 (1.40 to 1.66)	1.68 (1.56 to 1.80)	8%	12%
Diuretic use, %	10.8 (9.6 to 12.0)	8.3 (7.5 to 9.1)	2.5 (1.1 to 3.9)	2.02 (1.61 to 2.54)	4.39 (3.53 to 5.47)	10%	14%
CKD: eGFR <60 mL/min, %	12.0 (10.9 to 13.2)	7.5 (6.7 to 8.2)	4.6 (3.2 to 6.0)	2.23 (1.77 to 2.81)	3.96 (3.14 to 5.00)	27%	25%

[a]For dichotomous variables (Education, Poverty, Diuretic Use, and CKD), the data have been scored 0 for “no” and 1 for “yes”; therefore, the mean is equivalent to the proportion.
[b]Adjusted for age and race; [c]Difference in a dichotomous factor or 1 SD difference in a continuous factor.
BMI=body mass index; CKD=chronic kidney disease; DASH=Dietary Approaches to Stop Hypertension; eGFR=estimated glomerular filtration rate; SD=standard deviation
*Estimates for each risk factor generated from sample of men with complete data on Gout status, risk factor of interest, and all other factors. Risk Factor-Outcome Associations adjusted for age and self-reported race.

© 2022 McCormick N et al. *JAMA Network Open*.

eFigure. Age-Adjusted Prevalence of Gout Among Black and White US Adults Between NHANES III (1988-1994) and NHANES 2006-2017

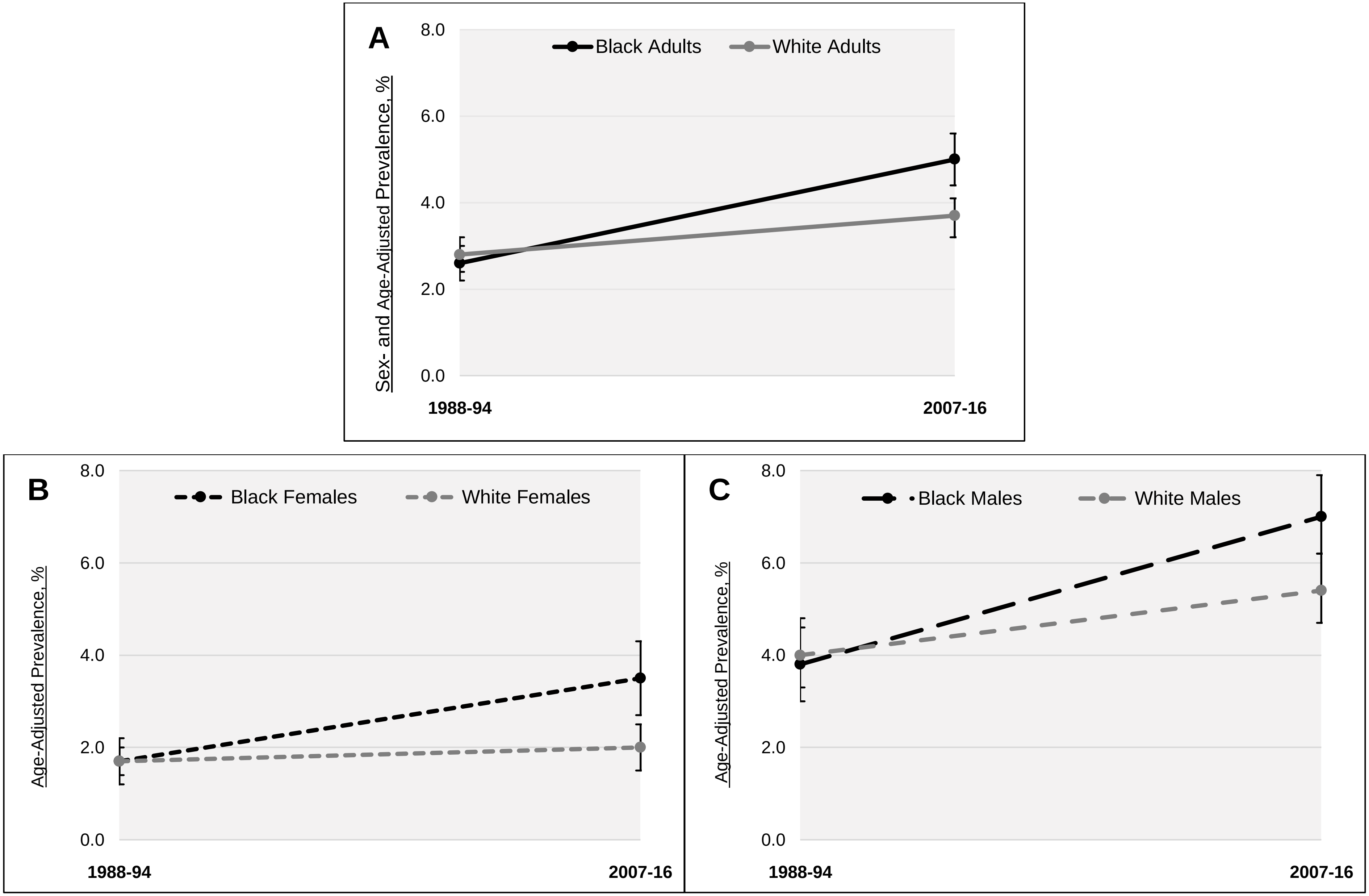

(A) Black and White adults overall; (B) Black and White women; (C) Black and White men.

© 2022 McCormick N et al. *JAMA Network Open*.
